# Physicochemical Characterization and Cytotoxicity Screening of a Novel Colloidal Nanogold-Based Phenytoin Conjugate

**DOI:** 10.3797/scipharm.1402-03

**Published:** 2014-08-21

**Authors:** Susan Cleave A Suneetha, Bala Praveen Chakkravarthy Raghupathy, P. K. Suresh

**Affiliations:** ^1^ School of Bio Sciences and Technology, VIT University, Vellore - 632014, Tamil Nadu, India.; ^2^ Centre for Nanotechnology Research, VIT University, Vellore - 632014, Tamil Nadu, India.

**Keywords:** Phenytoin, Colloidal gold, *In vitro* release, Drug delivery

## Abstract

A novel, colloidal nanogold-based drug delivery system for phenytoin, a well-known anti-epileptic drug with an enhanced efflux via P-glycoprotein, has been proposed in this study. The vital physical properties that would aid in predicting the biological interaction of this system were profiled using various techniques such as UV-Vis, DLS, and TEM in corroboration with theoretical calculations. It was significant to note that the binding of phenytoin to colloidal nanogold was strongly pH-dependent with the optimum at pH 5.5 and a consistently reproducible spectral shift. Analysis of the conjugate by FTIR revealed that the imide functional group of phenytoin mediated a dative coordinate bond to colloidal nanogold at the optimum pH. The amount of the drug bound to the gold was quantified to be 85.8±2.5% (w/v) by HPLC. Hypothetically, the surface charge of the conjugate could possibly imply charge-mediated uptake across the cell membrane. Further, the novel conjugate was screened for its cytotoxicity in two cell lines and the dosage range was identified. Subsequent development, thorough evaluations in suitable model systems, and the potential for bioimaging to track the payload would validate our hypothesis on the conjugate for better intracellular retention at the site of action, and thereby achieve the targeted delivery.

## Introduction

Nanoparticles are widely studied for biomedical applications such as drug delivery, diagnostics, and *in vivo* imaging. When nanoparticles are functionalized or linked to agents such as drugs, ligands, image contrast compounds through covalent linkages like amide or disulphide bonds, or through methods such as encapsulation, surface attachment or entrapment, they can be targeted towards a certain therapeutic application. For drug delivery through nanoparticles, advantages such as increased aqueous solubility, prolonged release, improved bioavailability, and decreased toxic side effects of the drug can be achieved [1]. Of the many nanoparticles emerging, gold nanoparticles have gained tremendous importance, especially in applications such as drug delivery, bioimaging, single molecule tracking, and biosensing due to some of its inherent properties [2].

The physicochemical properties of gold nanoparticles such as small size, large surface area to mass ratio, high surface reactivity, and the presence of surface plasmon resonance (SPR) bands along with their unique properties like their inert core, bio-compatibility, and less toxicity makes them a suitable agent for drug delivery [3]. The ease of synthesizing gold nanoparticles in varying core sizes, ranging from 1 nm to 100 nm is an added advantage. An important aspect that needs to be considered and understood well for an effective drug delivery system is the optimization of the charge, size, and surface functionality of the nanoparticles, since these variables, in major part, will strongly influence not only the nature of their interaction with the receptor(s), but also their uptake across anatomical and/or physiological barriers [4].

Phenytoin (5’,5’-diphenylhydantoin) is an anti-epileptic drug for the treatment of tonic clonic (grand mal) or partial seizures and for the acute treatment of generalized status epilepticus [5]. At the cellular level, the action of phenytoin prolongs the inactivation of voltage-activated sodium ion channels and reduces the firing of neurons at high frequencies [6]. With a high melting point of 295 to 298°C, phenytoin is a crystalline compound having strong intermolecular hydrogen bonding. It has been reported to have poor bioavailability and is classified as a WHO essential drug of Biopharmaceutical Classification Class II which is practically insoluble with a calculated logP of 2.09 and logP of 2.14 (Hazardous Substances Data Bank) [7].

In this study, phenytoin was conjugated to colloidal nanogold and its cytotoxicity was screened in two cell lines. This is the first study of its kind to bind phenytoin to colloidal nanogold (referred to as AuPht), to characterize its physicochemical parameters with a battery of techniques and to screen its cytotoxicity. AuPht, when sufficiently evaluated and validated in model biological systems, using an appropriate route of drug administration, can be aimed to enhance the poor bioavailability of phenytoin based on the advantages that nanotechnology provides for the advancement of therapeutic applications, especially to overcome the challenging drug resistance caused by the P-glycoprotein drug efflux.

## Experimental

### Materials

Chloroauric acid was purchased from the Sisco Research Laboratories (India), diphenylhydantoin from Sigma Aldrich (India), trisodium citrate from Qualigens Fine Chem (Mumbai, India), sodium hydroxide from SD Fine, India, and potassium dihydrogen phosphate from HiMedia. Phenazine methosulfate (N-methylphenazonium methosulfate), fetal bovine serum, L-15 (Leibovitz) cell culture medium (with L-glutamine) and MEM (minimal essential medium) with Earle’s salt, NEAA, and L-glutamine were purchased from HiMedia Laboratories Pvt. Ltd. (India). XTT (2,3-bis(2-methoxy-4-nitro-5-sulfophenyl)-5-[(phenylamino)carbonyl]-2*H*-tetrazolium hydroxide) was bought from Sigma Chemical Co. (St. Louis, MO, USA). HPLC grade methanol and water were used as procured; 0.45 μm and 0.2 µm nitrocellulose membranes from Sartorius were used for filtration.

### Synthesis of Colloidal Gold

The classical citrate reduction method was adapted to synthesize the colloidal gold based on the procedure described in [8]. Fifty (50 ml) 0.01% chloroauric acid was brought to boil in a round-bottom flask fitted with a condenser under continuous stirring. One and three-fourths of a ml (1.75 ml) of freshly prepared 1% trisodium citrate solution was rapidly added. When a final ruby red color appeared, boiling was continued for 15 min after which the stirring continued for another 15 min. After cooling to room temperature, it was stored in a brown bottle at 4°C.

#### Theoretical Size and Concentration Determination of Colloidal Gold

The surface plasmon resonance (SPR) of the colloid was verified by a UV-visible spectrum obtained in the Cary 50 UV-visible Spectrophotometer (Varian Inc., Australia). Since the optical properties of colloidal gold are dependent on size and wavelength, the information derived from its surface plasmon can be used to identify its size. Based on the good agreement presented by the mean free path corrected Mie theory and experimental data, equations to calculate the size and concentration of colloidal gold were given by Haiss *et al* [9]. Following are the equations used for determining the size and concentration:


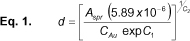


Where A_spr_ is the absorbance at the peak SPR, c_Au_ (moles/l) is the amount of gold used in the synthesis, C_1_ = −4.75, and C_2_ = 0.314. The error calculated by Haiss is ~ 6%.

The concentration of the colloid using equation (2) in nps/ml (nanoparticles per ml) is first calculated using the known absorbance at 450 nm based on which further concentration calculations can be done.

Subsequently, the concentration in µg/ml was calculated.


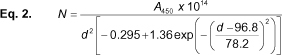


Where N is the number density of colloidal gold in nps/ml, A_450_ is the absorbance at 450 nm, and d is the diameter (in nm) of colloidal gold.

Using N, the concentration (Wt. con) in µg/ml (ppm) was calculated as follows:


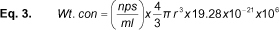


Where r is the radius of AuNPs in nm.

#### Percentage Yield of Colloidal Nanogold

In order to experimentally determine the concentration of the synthesized colloidal nanogold, a known volume of the colloid was acid-digested with 7% conc. HNO3. After complete dissolution, the resulting solution was analyzed with atomic absorption spectroscopy (Varian 240) at the emission wavelength of 267 nm (N=3). A calibration curve from 0 ppm to 60 ppm was used to calculate the colloidal nanogold yield obtained based on the following formula:


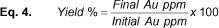


### Formulation of the Colloidal Nanogold-Phenytoin Conjugate

A known amount of phenytoin (1 mg) was added to 10 ml of colloidal nanogold and the suspension was stirred vigorously at room temperature. The color changed from red to blue in about 8 hours. The SPR changes observed with time, implying the conjugation, were monitored through a UV-visible spectrum. The conjugate, AuPht thus formed, was characterized by dynamic light scattering (DLS) and high resolution transmission electron microscopy (HRTEM).

#### Binding Kinetics of Phenytoin to Colloidal Nanogold with Respect to Time and pH

The binding of phenytoin to colloidal gold was studied with respect to time and pH. The changes observed in the SPR were used to study the time dependence of the conjugation process by taking spectral measurements every hour. Also, by varying the pH in the range of 5 to 7, the binding was studied in the first hour.

#### Dynamic Light Scattering

The particle size and the polydispersity index of the colloidal nanogold and AuPht were determined (N=2). The dynamic light scattering experiments were performed in the Brookhaven 90Plus Particle Size Analyzer (software ver. 5.31) (Brookhaven Instruments Corporation, UK). This method provides an accurate measure of the size and the monodisperse condition of AuPht

#### Zeta Potential Analysis

The stability of the colloidal nanogold phenytoin conjugate was studied by determining its zeta potential at different pHs. Further, in order to gain an understanding of its behaviour in more physiologically relevant conditions, its zeta potential in PBS, L-15, and MEM cell culture media (without serum) was determined at 25°C (N=2). In all the three scenarios, the pH was maintained at 7.4

#### High-Resolution Transmission Electron Microscopy (HRTEM)

The exact size and morphology of the synthesized colloidal gold and AuPht were determined by the transmission electron microscope (TEM) analysis. The samples were dropped onto the carbon-coated copper grids and vacuum dried. They were then observed under a JEOL 3010 TEM at 300 kV.

#### FTIR

Pure phenytoin and lyophilized AuPht were prepared into pellets by mixing with KBr. They were then analyzed by the Perkin Elmer Spectrum1 FTIR spectrometer at a resolution of 1 cm^-1^ at a scan range of 450 to 4000 cm^-1^. The FTIR of the conjugate suspended in the above-mentioned physiologically relevant media incubated at 37°C for 24 hours at pH 7.4, was also performed to verify the interaction of phenytoin with the colloidal nanogold.

#### Phenytoin Binding Efficiency to Colloidal Nanogold

The HPLC method was adapted based on Varaprasad et al. [10]. The samples were analyzed in an isocratic HPLC (Waters Corp.) with a C18 column (Symmetry 5 µm, 4.6 x 150 mm). Twenty (20 μl) of the sample was injected using a Rheodyne injection syringe at a flow rate of 0.7 ml/min, maintaining the column at 25°C. Freshly prepared methanol: 0.05 M and phosphate buffer (pH 2.8) (60:40 v/v) were used after degassing and filtering through a 0.45 μM filter. The UV detection wavelength was identified to be 258 nm (Waters 2487 Dual Absorbance Detector). Based on these conditions, the amount of phenytoin bound to the colloidal nanogold was quantified. AuPht (10 ml) was centrifuged and the pellet obtained was resuspended in the mobile phase. Standards in the range of 0.1 mg/ml to 0.3 mg/ml were prepared with the mobile phase as diluents (N=2). Colloidal nanogold was used as a control and analysed under the same conditions. In order to cross-verify the amount of unbound phenytoin, the supernatant was also analysed.

#### Cell Lines and Maintenance

Caco-2 (human colorectal adenocarcinoma) and MDA-MB-435s (human breast carcinoma) were purchased from the National Centre for Cell Science (Pune, India). Caco-2 cells were maintained in minimum essential medium (MEM) (Eagle) with non-essential amino acids (with 5% CO_2_) [11] and MDA-MB-435s cells in L-15 (Leibovitz’s) medium [12]. Both the cell lines were maintained with 10% fetal bovine serum in a humidified atmosphere at 37°C. The cells were maintained in their growing phase at 70% confluency with regular passaging.

#### Cytotoxicity Assessment

Phenytoin, colloidal nanogold, and AuPht were tested for cytotoxicity by the XTT-formazan dye formation assay [12, 13]. Caco-2 and MDA-MB-435s were seeded in their respective culture media (200 µl, 2 x 10^4^ cells/well and 1 x 10^4^ cells/well, respectively) in 96-well plates and incubated at 37°C for 24 hours without/with CO_2_. After incubation, the control wells were replenished with fresh medium and the test wells were treated with different phenytoin concentrations (20, 10, and 5 µg/ml). The cells were further incubated for 24 hours under the same conditions. After the treatment, the medium in each well was replenished with 200 µl of fresh medium followed by 50 µl of XTT (0.6 mg/ml containing 25 µM phenazine methosulphate). The plate was then incubated for another 4 hours under the same conditions after which the absorbance was measured at 450 nm (with a 630 nm reference filter) in a Dynex Opsys MRTM Microplate Reader (Dynex Technologies, VA, USA).

Based on the known therapeutic index of phenytoin of 10–20 µg/ml [6], concentrations of phenytoin were set at 20, 10, and 5 µg/ml for initial dose-finding experiments. Similarly, range-finding experiments (2.5 µg/ml to 40 µg/ml; N=3) were performed for the selection of non-cytotoxic doses of colloidal nanogold. Since concentrations higher than 10 µg/ml were extraordinarily toxic, they weren’t chosen. Based on phenytoin concentrations determined from HPLC for AuPht, 20, 10, and 5 µg/ml were calculated and used for treating the cells.

The percentage of cytotoxicity was calculated by the following formula:


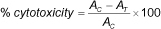


Where A_c_ is the absorbance of the control wells and A_**T**_ is that of the treated wells. Data were expressed as the mean±SD from three determinations. Statistical analysis was performed using Student’s t-test (two-tailed), with P < 0.05 as the criterion of significance.

## Results and Discussion

### Synthesized Colloidal Gold

**Fig. 1.**
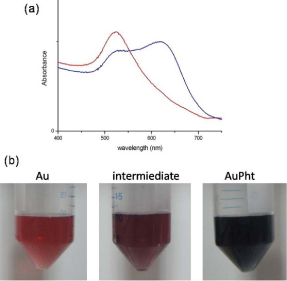
(a) The UV-visible spectrum of colloidal nanogold (red) and the blue shift observed due to the conjugation of phenytoin with colloidal nanogold (blue); (b) The colour changes observed in the conjugation process

**Fig. 2.**
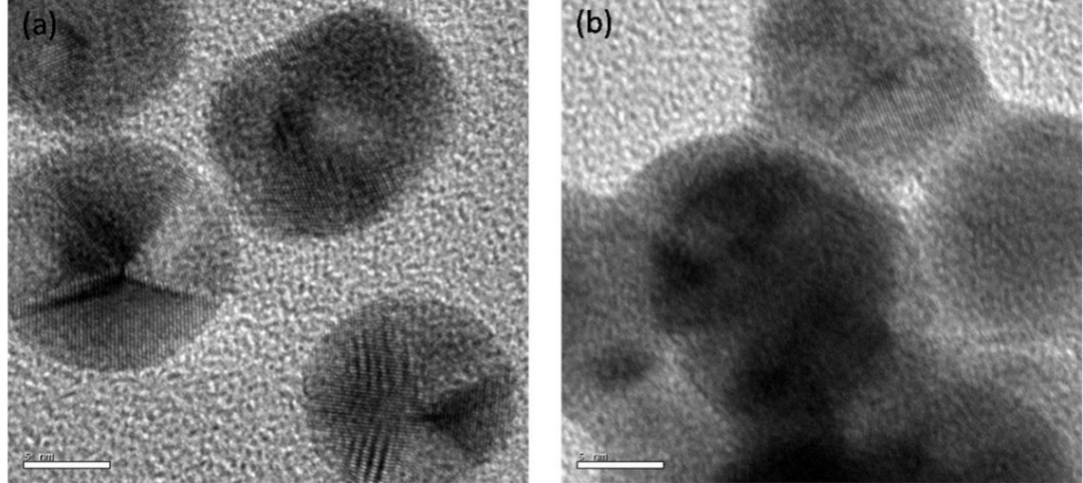
HRTEM images showing (a) the spherical shape of colloidal nanogold having a of diameter 13 nm and (b) the almost spherical shaped AuPht of diameter 40 nm; Scale bar – 5 nm

The wine red color along with the single absorption peak at 521 nm in the UV-visible spectrum indicated the presence of spherical gold particles in the colloid (Fig. 1a). The theoretical size was calculated to be 12.9 nm using the absorbance maxima in equation 1. The DLS experiments gave an average particle size of 15±2 nm with a polydispersity index of 0.32±0.03. The HRTEM analysis confirmed the presence of spherical particles having the nano-size range of 13 nm in the colloid (Fig. 2a). Based on this, the surface area-to-volume ratio was calculated to be 0.46. Further, the yield of colloidal gold was calculated to be 87.9% and the concentration was 46.6±0.40 ppm, based on AAS data. This information was further validated by theoretical calculations using equations 2 and 3 and the concentration was calculated to be 47.1 ppm. The zeta potential of the colloidal nanogold was identified to be -73.0 mV, implying its high stability.

In order to achieve efficient delivery at the target site, the primary and equal importance should be given to the identification and characterization of the physical parameters of the system such as the surface-to-volume ratio, size, shape, and charge [14]. Alongside achieving size and shape control, the in-house synthesized colloidal nanogold was also uniformly dispersed in addition to obtaining a good yield and concentration. Next, the phenytoin conjugation was carried out.

### Colloidal Gold Phenytoin Conjugate

After phenytoin addition to the colloidal nanogold, the wine red color changed to blue, which was also indicated by a blue shift to a longer wavelength of 618 nm in the UV-visible spectrum (Fig. 1a). The visually observed color change was correlated with a shift in the λ_max_, and this, in turn, was correlated with the observed aggregation in the AFM images (data not shown).

Based on the intercoupling effect [15], the second peak arising at longer wavelengths can be attributed to the dipole plasmon resonance of the colloidal nanogold. The shift to the longer wavelengths was also accompanied by the weakening of the first peak implying a reduced interparticle spacing, thereby indicating conjugation and thus, the blue color. Some of our preliminary experiments with the cyclic voltammetry (data not shown) have verified the binding of phenytoin to the bare gold electrode: there was a reduction in the redox current conducted when standard potassium ferricyanide solution was used as the electrolyte.

The size of AuPht determined in the DLS experiments was 55 nm, while in HRTEM, it was 40 nm (Fig 2b). This size difference, albeit negligible, can be attributed to the fact that DLS measures suspended particles that are in continuous motion and therefore, possess an outer hydration shell. This shell contributes to the additional diameter that was obtained in DLS. Thus, we could confirm the conjugate as being irregular to spherically-shaped aggregates sized around 40 nm (Fig 2b). After 72 hours, it was observed that the aggregates agglomerated and they settled to the bottom.

The zeta potential for AuPht was found to be -44.6±1.56 mV (in water). By varying the pH, it was observed that the zeta potential attained zero at pH 1.2. Further from Table 1, the zeta potentials for the conjugate under physiologically relevant conditions in different media are reported. These values tend to be less negative or become positive when they are in circulation and interact with the serum albumin proteins that are present in the blood [16]. In regard to the fact that cellular surfaces are negatively charged due to the presence of glycosaminoglycans, it can be hypothesized that the uptake of AuPht could possibly be charge-mediated across the membrane [17].

**Tab. 1.** Zeta potential of the colloidal gold phenytoin conjugate in different physiologically relevant media

Medium	pH	Zeta potential (mV)
PBS	7.4	−9.95±2.05
L15	7.4	−5.7±0.57
MEM	7.4	−7.9±1.27
AuPht (water)	7.4	−50.55±0.21
AuPht (water)	6.7	−44.6±1.56

Kinetics of Phenytoin Binding with Respect to Time and pH

**Fig. 3.**
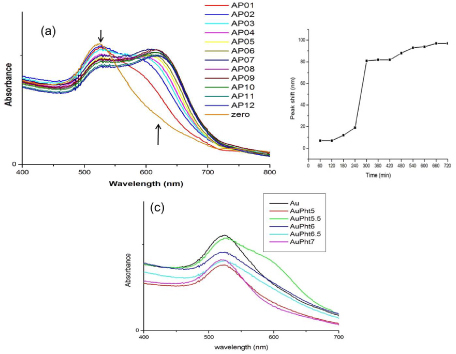
(a) Time-dependent binding kinetics of phenytoin to colloidal nanogold taken every hour for a period of 12 hours (N=3). The peak at 521 nm decreases as the new peak at 618 nm gradually appears. The absorbance of the peak at 618 nm shows no further increase beyond 8 hours, implying the conjugation process to be finished. (The numerals in the legend correspond to the time interval in hours and their respective colored spectral line); (b) A plot of the change in λ_max_ (∆λ_max_) with respect to time. The ∆λ_max_ can be observed to become constant beyond 8 hours indicating that the final λ_max_ is 618 nm; (c) Spectrum for pH-dependent binding of phenytoin to colloidal nanogold. The spectrum for pH 5.5 (labeled as AuPht5.5 and in green colour) shows the growth of an extra peak around the 600 nm region indicating the conjugation process to have started. The spectral lines of other pH values do not show this extra peak

A kinetic experiment was performed to identify the effect of time on phenytoin conjugation with the colloidal nanogold at pH 5.5. In the spectrum (Fig 3a), the color changes at intervals of 60 min were recorded and with time a distinct, reproducible, blue shift from 521 nm to 618 nm was noted. It was observed that there was no further increase in absorption at 618 nm after 8 hours. This indicates that the Pht-binding process was finished, which may be due to saturation of the particle surface at this time point. The change in λ_max _(Δλ_max_) was plotted as a function of time to know the changes in the peak shift and this is represented in Fig 3b. It can be seen that there is no further increase in the shift and the λ_max_ (i.e., 618 nm) does not change after 8 hours.

AuPht formation was also identified to be dependent on the initial pH of the colloid nanogold. The Fig. 3C shows the spectra at 1 hour after the addition of phenytoin at various pHs ranging from 5.0 to 7. No change can be seen in the absorbance maxima at pH 5.0, 6.0, 6.5, and 7.0, implying the dependency on the initial pH 5.5 of the colloidal nanogold.

This phenomenon can be reasoned based on one or more of the groups available for binding in the colloid. It has been identified that [Au^+^Cl^-^OH^-^]^-^ is the predominant gold species present in the pH range of 5.4 to 6.4 [18] of the synthesized colloidal nanogold. These species are formed in the mild acidic medium as a result of the citrate reduction of gold ions. Thus, it is these species that would be the most likely ones to interact with the functional groups of phenytoin.

Nature and Efficiency of Phenytoin Binding to Colloidal Gold

**Fig. 4.**
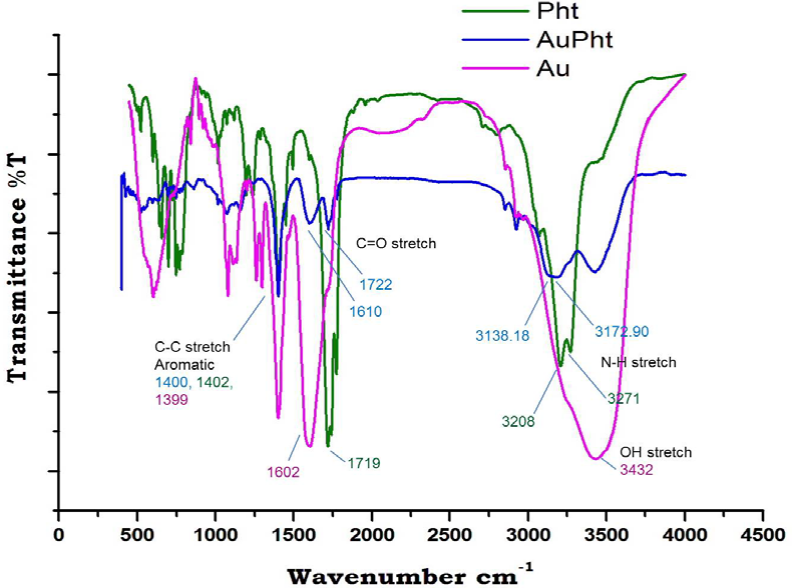
. FTIR spectrum showing the possible interaction of phenytoin with colloidal nanogold. The AuPht spectrum shows the shifted wave numbers in the NH stretch region in comparison to pure phenytoin (Pht). The spectrum of colloidal nanogold (Au) as well as that of unconjugated phenytoin is also shown as a reference. The corresponding spectral wave numbers are indicated in their respective colours along with each functional group

The FTIR spectrum of AuPht was studied at the 8 hour time point. The spectrum (Fig. 4) of pure phenytoin shows sharp stretching vibrations at 3271 and 3208 cm^-1^ for the NH group and at 3068 cm^-1^ for the aromatic C-H. Stretching vibrations for the carbonyl group of the hydantoin were observed at 1719 and 1740 cm^-1^. The peaks at 1402 cm^-1^ indicate the C-N stretch and those at wave numbers 746, 697, and 655 cm^-1^ indicate the C-H out of plane vibrations of the phenyl ring. This spectrum, when compared to that of AuPht, shows an overlap with the C-H (aromatic 2926 and phenyl ring 760 and 698.23 cm^-1^), C-N (1396 cm^-1^), and C=O (1722 cm^-1^) vibrations while only the NH stretching vibrations shifted to 3172.90 and 3138 cm^-1^ [19]. With reference to the FTIR of colloidal nanogold, the C=O vibrations (1602 cm^-1^) formed due to the citrate layer show a small difference in the corresponding wave number (1610 cm^-1^) in AuPht. This implies the presence of citrate in the conjugate which results in the negative charge.

**Fig. 5.**
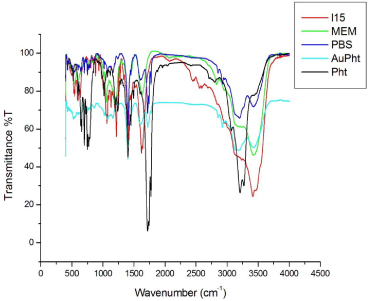
FTIR spectrum of AuPht in different biological media taken after a 24-hour incubation period at pH 7.4, in comparison with pure phenytoin (Pht). L15, MEM, and PBS are the three different media studied. The prevalence of the NH shift (3000–3250 cm^-1^) in AuPht after the 24 hours incubation indicates the conjugate’s stability in different media

This implies that the imide functional group of phenytoin mediates the interaction with the colloidal nanogold. Based on the existing reports of the nature of the colloidal gold [Au^+^Cl^-^OH^-^]^-^ [18] and from the interaction seen in our studies, it can be said that the lone pair of electrons from the nitrogen in phenytoin could possibly form a coordinate bond with the positively charged gold atom in the colloid. The above interaction was found to prevail when the conjugate was incubated with cell culture media and PBS at pH 7.4 at 37°C (Fig. 5). This implies the stability of the conjugate in the physiological media.

Based on the mentioned HPLC conditions, phenytoin from AuPht was eluted at approximately 7 min. With reference to the standard pure phenytoin concentrations, it was identified that 85.8±2.5% of the added drug was bound to the colloidal nanogold.

Earlier studies have shown that the nitrogen atom from the functional group of the drug mediates the binding between the drug and the gold nanoparticles. In the case of Ciprofloxacin [20], a hydrophilic drug, the nitrogen atom from its imino moiety was bound to gold nanoparticles. Similarly, the amino groups of streptomycin, gentamycin, and neomycin [21] aided binding to gold nanoparticles. Doxorubicin, a hydrophobic drug, with broad antitumor activity, also binds to negatively charged gold nanoparticles in the acidic conditions through the amine groups [22].

### Cytotoxicity Assessment

**Fig. 6.**
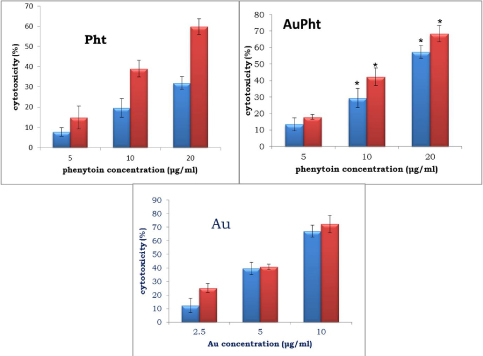
XTT-cytotoxicity results of phenytoin (Pht), AuPht, and colloidal nanogold (Au) in Caco-2 (red) and MDA-MB-435s (blue) cells at different concentrations over a time period of 24 hours. Results are represented as the mean ± SD. Asterisks indicate significant differences (p ≤ 0.05, N = 3)

Using the XTT-cytotoxicity assay, the effect of AuPht on cell viability was studied on Caco-2 and MDA-MB-435s cells. It was observed that phenytoin, at 20 µg/ml, showed a cytotoxicity of 31.88±3.2% and 59.75±3.8% in MDA-MB-435s and Caco-2 cells, respectively (Fig. 6). The corresponding cytotoxicity of AuPht for the same phenytoin concentration was 57.43±3.9% and 68.4±4.9% in the MDA-MB-435s and Caco-2 cells, respectively, as seen in Fig. 6. The colloidal nanogold at 10 µg/ml showed high cytotoxicity of 66.18±4.5% and 72.35±6.4% in MDA-MB-435s and Caco-2 cells, respectively.

AuPht showed higher cytotoxicity than pure phenytoin, which might be due to the citrate as well as other ions that the colloid is composed of, which aid in providing stability to the colloidal nanogold. This aspect notwithstanding, pure phenytoin, colloidal nanogold, and AuPht expressed relatively higher cytotoxicity in the Caco-2 cell line when compared with the results obtained in the MDA-MB-435s cell line. This may be attributed to the differences in the receptors they possess and whether P-gp could be a possible reason, which needs further studies. Caco-2 is an intestinal carcinoma cell line which is known to highly express P-gp and MDA-MB-435s is a cell line that does not express P-gp, even though it is permeable to the tested nanoconjugate. Caco-2 is the well-known cell line which is used to screen compounds for their intestinal permeability.

From previous studies reported by other groups worldwide, surface charge affects the biodistribution of colloidal nanogold such that positively charged particles accumulate more in the kidneys, while negative and non-charged particles show a higher accumulation in the liver [23], thereby providing corroborative data for the plausible electrostatic interaction-mediated uptake in our cell-based system. It was also shown that the toxicity of nanogold is influenced by the surface charge. Anionic nanogold demonstrated non-toxicity, while cationic nanogold showed moderate toxicity, which resulted from its interaction with the cell membrane [24, 25].

Based on the different reported studies, we can say that the size [26] and surface charge of colloidal nanogold and the novel AuPht will play a major role in their biodistribution and subsequently their mode of intracellular trafficking, as well as clearance mechanisms, subsequent to their pharmacological actions at the target site. Moreover, our results warrant a thorough evaluation of its uptake (relative contributions of diffusion/concentration gradient-dependent mechanism vs. charge-mediated uptake) in cell-based model systems to better delineate the mechanism(s). This would also help in studying the nature of AuPht in the dispersed medium.

Further, in the future evaluations of the conjugate in suitable model systems as a possible means to achieve better retention of phenytoin at the target site of action, this approach would provide an added advantage of tracking the movement of AuPht, due to the inherent fluorescent properties of colloidal nanogold. More studies focusing on the *in situ* conjugation methods may serve to further validate our approach for colloidal gold-based drug delivery. Moreover, our results warrant a thorough evaluation of its uptake (relative contributions of the diffusion/concentration gradient-dependent mechanism vs. charge-mediated uptake) in cell-based model systems to better delineate the mechanism(s). This would also help in studying the nature of the conjugate in the dispersed medium. These studies will help in making further improvements in the design and delivery of phenytoin, incorporating cleavable linkers in the AuPht conjugate.

## Conclusion

In this study, we have formulated the novel colloidal nanogold phenytoin conjugate. With the aid of various analytical techniques corroborated with theoretical calculations, major physical properties such as size, shape, and charge of the colloidal nanogold and the conjugate were evaluated. The lone pair of electrons of the nitrogen in the imide functional group of phenytoin mediated a coordinate bond with the positively charged gold atoms in the colloid. A good amount of the drug, 86%, was identified to bind with the colloidal nanogold. The zeta potential in the physiological conditions, hypothetically, implies that the uptake of the conjugate would be charge-mediated across the cell membrane. Further, the novel conjugate was screened for its cytotoxicity in two cell lines and a dose range was identified that has to be optimized in future applications. When combined with the ability to track the payload, the colloidal nano-gold phenytoin conjugate can have potential applications in the field of targeted drug delivery after thorough evaluation of its safety and biocompatibility.
